# Analysis of Topological Parameters of Complex Disease Genes Reveals the Importance of Location in a Biomolecular Network

**DOI:** 10.3390/genes10020143

**Published:** 2019-02-14

**Authors:** Xiaohui Zhao, Zhi-Ping Liu

**Affiliations:** Department of Biomedical Engineering, School of Control Science and Engineering, Shandong University, Jinan 250061, Shandong, China; zhaoxiaohui199612@163.com

**Keywords:** disease gene, network topological parameter, location in network, systems biology

## Abstract

Network biology and medicine provide unprecedented opportunities and challenges for deciphering disease mechanisms from integrative viewpoints. The disease genes and their products perform their dysfunctions via physical and biochemical interactions in the form of a molecular network. The topological parameters of these disease genes in the interactome are of prominent interest to the understanding of their functionality from a systematic perspective. In this work, we provide a systems biology analysis of the topological features of complex disease genes in an integrated biomolecular network. Firstly, we identify the characteristics of four network parameters in the ten most frequently studied disease genes and identify several specific patterns of their topologies. Then, we confirm our findings in the other disease genes of three complex disorders (i.e., Alzheimer’s disease, diabetes mellitus, and hepatocellular carcinoma). The results reveal that the disease genes tend to have a higher betweenness centrality, a smaller average shortest path length, and a smaller clustering coefficient when compared to normal genes, whereas they have no significant degree prominence. The features highlight the importance of gene location in the integrated functional linkages.

## 1. Introduction

Complex diseases such as neurodegenerative disorder, metabolism syndrome and cancer are often hypothesized as the results of molecular perturbations and dysfunctions [1,2,3]. In systems biology and medicine, the onset of complex diseases is considered to be the consequence of abnormal interactions among multiple genes, gene products, and metabolic compounds [3,4]. The disease genes play a driving role, causing locally original dysfunctions, and then signaling pathways spread their affections and cause global misalignment, even leading to mortality [5]. The disease genes provide causal information of the dysfunctional occurrence and development.

With the screening of high-throughput technologies, more and more putative disease genes have been identified [6]. For instance, the genome-wide association study (GWAS) provides a systematic investigation of genetic variants in case-control population individuals to see if any variant in the genome is associated with a particular trait [7]. As we all know, the papers about biomedicine and health have been documented in the national library of medicine [8]. There is summary statistics about the most popular genes studied in these papers. It is found that the most popular genes are all related to complex diseases in oncology and immunology [9].

A network provides a mathematical framework for deciphering the relationship between biomolecules [10,11,12]. In which, nodes refer to the biomolecules and edges refer to their relationships. The genes and their products perform their normal or dysfunctional roles by interacting with each other exactly in the form of a network [4,13]. Due to recent rapid increase in biomolecular interaction data, the study on biomolecular networks has been booming [14,15]. For instance, the number of protein interactions deposited in the STRING database has grown up to 1380 million in thousands of organisms [16]. In a network, its topological parameters define the quantitative patterns and measures of the nodes and edges [14]. For instance, the degree of a node refers to the number of edges incident to the node. The topological properties, such as degree and its distribution, provide detailed descriptions of the network’s features. Thus, the network’s localization and organization are reflected in its topological parameters.

The topological parameters also characterize the pattern of genes in a networked system [14]. There are some examples that the topological prominence implies biological essentiality in networks [17,18,19]. For instance, the hub genes often refer to the nodes with high degree in a network [20]. Interestingly, it has been proved that disease genes are often not hub genes for their functional importance in the network structure [21,22]. The non-conventional findings deepen our understanding about the network localization of disease genes. In a cell, an interactome often refers to the whole set of gene interactions [23]. The location of nodes in the interactome highly coordinate their specific dysfunctions during disease development and progression [24,25]. The interactome presents a map of functional layout among genes. The information flow transmitting from disease genes to normal genes are also determined by the local neighbors and environments [1,2]. Their location in the network is also the crucial determinant of dysfunctions from the systematic perspective. It is of paramount interest to explore these parameters of disease genes in the interactome.

In this paper, we focus our analysis on the topological parameters underlying disease genes on an integrated gene–gene interaction network. For generality, we firstly investigate several network topological properties of the top-ten popular disease genes. Four widely-used network properties (i.e., node degree, betweenness, clustering coefficient, and shortest path length) are identified for representing the network localization of disease genes. For specificity, we validate the observed features underlying these network properties in three typical complex diseases (i.e., Alzheimer’s disease (neurodegeneration-related), diabetes mellitus (metabolism-related), and hepatocellular carcinoma (cancer-related)). We collect their disease-related genes and verify the identified topological patterns from the top-ten most popular genes in the three types of diseases, respectively. The differences of the network characteristics are discovered between disease genes and other randomly-selected normal genes. The topological distinctiveness between disease genes and their neighbors also indicate the specificity of network location in the pathogenesis of disease. For checking the independence between the parameters, their pairwise correlations are identified. The analysis highlights the importance of location of disease genes in a network.

## 2. Materials and Methods

### 2.1. Disease Genes

Firstly, we accessed the most popular genes from the list of “hot studies” in all human genomes [9]. The top-ten genes have been investigated in more than 40,000 papers. Table 1 lists their details. Apparently, these genes are all highly related to complex diseases [26]. Some of them are disease causal mutations (e.g., *TP53* (related to cancers) and *APOE* (related to Alzheimer’s disease)). Some of them perform severe dysfunctions related to inflammation and abnormal phosphorylation in diseases (e.g., *TNF* and *AKT1*). For generality, we identified their topological parameters in our integrated biomolecular network.

For specificity and justification, we also verified the findings of the top-ten-popular genes in three other complex diseases (i.e., Alzheimer’s disease (AD), diabetes mellitus (DM), and hepatocellular carcinoma (HCC)). The disease genes of these complex diseases were composited from KEGG [27], GWASdb [6], and GWAS Catelog [8]. The number of disease genes for AD, DM, and HCC were 171, 46, and 168, respectively. They are available from Supplementary material S1.

### 2.2. Integrated Biomolecular Network

**Figure 1:**
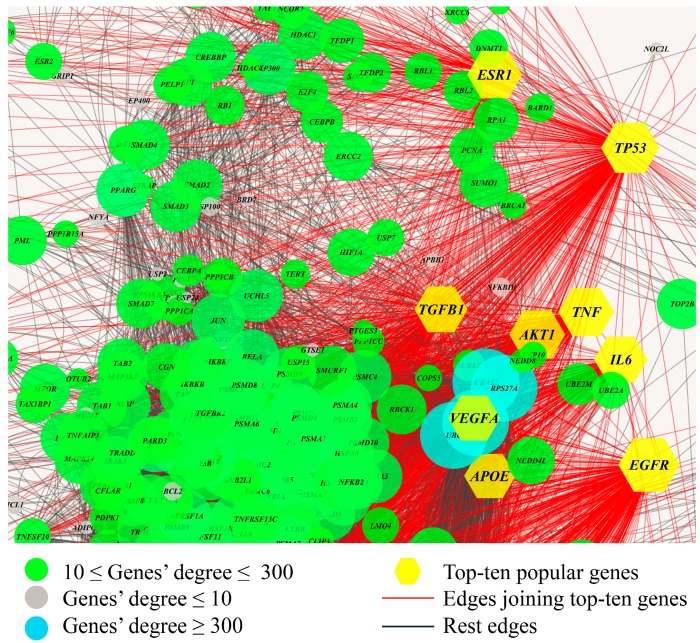
A partial view of the integrated biomolecular network. The meanings of the colors of the nodes and edges are shown in the legend. The nodes size is in proportion to the number of its neighbors.

For building up an interactome, we downloaded the documented biomolecular networks from STRING [16], BIND [28], MINT [29], BioGrid [30], IntAct [31], DIP [32], and HPRD [33]. In these databases, multiple types of biomolecular interactions are included in the integrated network. For completeness, the interactions referring to gene–gene co-expression, gene co-occurrence, gene fusion, gene regulation, and annotated pathways were all contained in the interactome. In total, the biomolecular network contained 7018 nodes and 224,127 edges. Figure 1 illustrates the global view of a typical part of the integrated gene–gene functional linkage network.

### 2.3. Network Topological Parameters

Some topological parameters have been defined to describe the properties of location in the network structure for quantifying their centrality or functionality [34]. For simplicity, we chose the four most robust measures of network topology to investigate the network properties [4].

#### 2.3.1. Node Degree

In a network, the degree of a node is defined as the sum of all edges connected to it [14]. If a node has a degree of *n*, it refers to *n* neighbor nodes connecting to it. Usually, the probability distribution of all node degrees is named as the degree distribution of the network. It is proved to be a power-law distribution in biomolecular complex networks [10,35,36].

#### 2.3.2. Average Shortest Path Length

In an unweighted network, the shortest path between two nodes *i* and *j* refers to the path between them with the smallest number of edges. The distance *d_ij_* between the two nodes refers to the shortest path between them. The average shortest path (network distance or network diameter) of an entire network is the average path length of all possible pairs of nodes [14,35], namely,

(1) $$L=\frac{2}{N\left(N+1\right)}\sum_{i\leq j,i,j\in G}d_{ij},$$

where *N* represents the number of nodes in the network *G(V*,*E*), node *i* and node *j* are in the network *G*. Here, the distance from a node to itself is defined to be zero.

#### 2.3.3. Clustering Coefficient

Clustering coefficient (CC) reflects the aggregation property underlying the nodes in a network [14], which refers to the tendency of gathering together of these nodes. In the network, CC depicts the average value of the ratio of the actual edge of a node in the complex network to all the possible edges, in essence,

(2) $$CC=\frac{n}{\left(\begin{array}{l} k\\ 2 \end{array}\right)}=\frac{2n}{k\left(k-1\right)},$$

where *n* is the number of edges connected to the node and its first-order neighbors, and $\left(\begin{array}{l} k\\ 2 \end{array}\right)$ is the number of its adjacent neighbors. The key of calculating CC is to obtain this value. We employed the node neighbor subgraph algorithm to achieve it from the adjacency matrix of the network. We found all neighbors of a location element in the adjacency matrix and got the number of edges. In an undirected graph, because of the symmetry of its adjacency matrix, the number of neighbors is two times of the number of edges. Therefore, the actual value should be divided by 2 [14].

#### 2.3.4. Betweenness Centrality

Based on shortest paths, betweenness centrality (BC) is a measure of centrality for assessing the importance of individual nodes in a network [14]. The BC value of a node is the ratio of all the shortest paths between the other nodes to the total number of these shortest paths that pass through the node, in essence,

(3) $$BC(v)=\sum_{i\neq \nu \neq j}\frac{\delta_{ij}(v)}{\delta_{ij}}$$

$\delta_{ij}$ is the number of the shortest paths from node *i* to node *j*. $\delta_{ij}(v)$ refers to the shortest paths from node *i* to node *j*, through node *v*.

### 2.4. Comparison Statistics

We compared the topological parameters of disease genes and the randomly-sampled same-size genes (no overlap between them). In accessing the statistical significance of their difference, we used the Mann–Whitney U test to obtain a *p*-value between the parameters in the two gene sets.

To assess the relationship between two topological parameters, we used the Spearman rank correlation coefficient to measure their association. Spearman rank correlation is a nonparametric measure of statistical dependence between two variables [37].

## 3. Results

### 3.1. The Values of Network Topological Parameters

**Figure 2:**
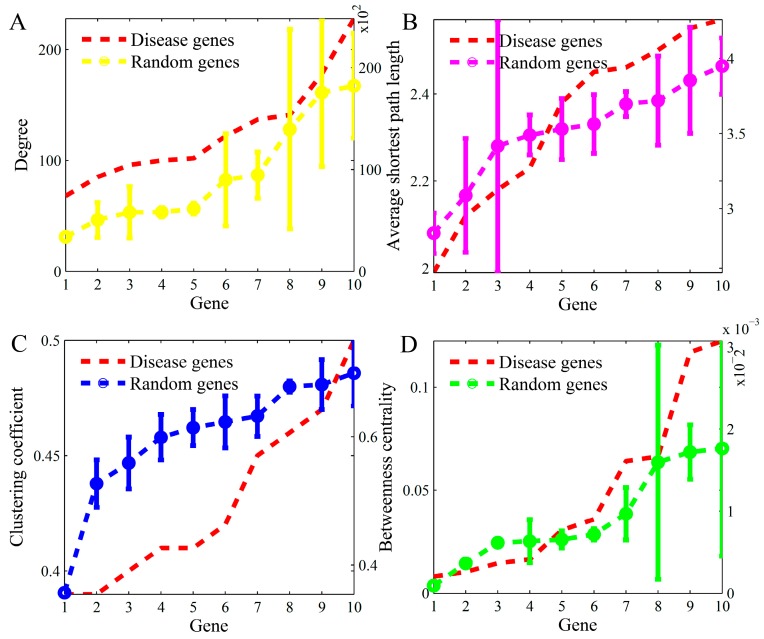
The comparisons of four topology parameters between disease genes and randomly-selected normal genes: (**A**) degree, (**B**) average shortest path length, (**C**) clustering coefficient, and (**D**) betweenness centrality. The x-axis is for ten genes, the left y-axis is for “mean” and the right y-axis is for “standard deviation”.

In the integrated biomolecular network, we identified the values of network property parameters for the top-ten popular disease genes respectively. Table 2 lists the values of their topological properties. For illustrating the specificities of parameters in these disease genes, we randomly chose ten other genes and calculated their topological parameters correspondingly. We repeated the random processes ten times. Figure 2 demonstrates the comparisons of the four parameters in the popular-studied genes with those in the same number of randomly-selected genes. In each subfigure, the genes were sorted based on the values of the parameters.

As shown in Figure 2, we found that the degree and BC of disease genes were, on average, higher than those of random genes. Whereas the average shortest path length and CC of disease genes were, on average, lower than those of random genes. In fact, degree is also a kind of centrality measure referring to the centrality of node in a network. The higher centrality of degree and betweenness indicated the crucial roles of genes in the network. Moreover, the lower average shortest path length also proved the importance of disease genes. Interestingly, the CC of random genes was generally higher than those of disease genes. This means that disease genes did not tend to be the central genes in network modules. The results provide evidence that the disease genes were located in critical positions in the network. Their locations reflect their functional importance in the interactome.

**Figure 3:**
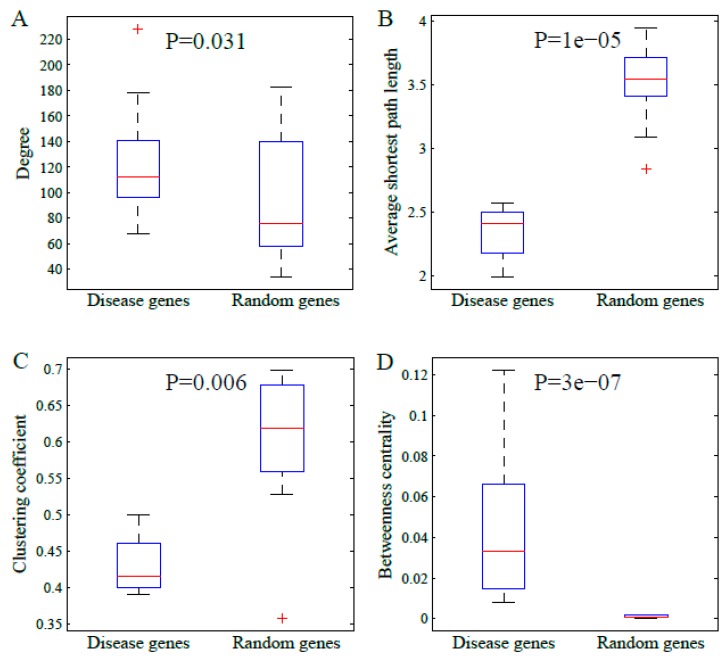
The boxplots of the topological parameters in disease genes and groups of random genes: (**A**) degree, (**B**) average shortest path length, (**C**) clustering coefficient, and (**D**) betweenness centrality. The *p*-value in each subfigure refers to the statistical significance of difference via Mann-Whitney U-test.

We also certified the comparison results in larger number of random samplings. We randomly selected ten groups of genes with ten genes per group, respectively. Then, the four topological parameters were calculated. The results are shown in Figure 3. The comparisons illustrated that the disease genes tended to have a higher degree and betweenness centrality, but they also tended to have smaller average shortest path length and clustering coefficient. These are consistent with those shown in Figure 2.

### 3.2. The Parameters in the First-Order Neighbour Genes

**Figure 4:**
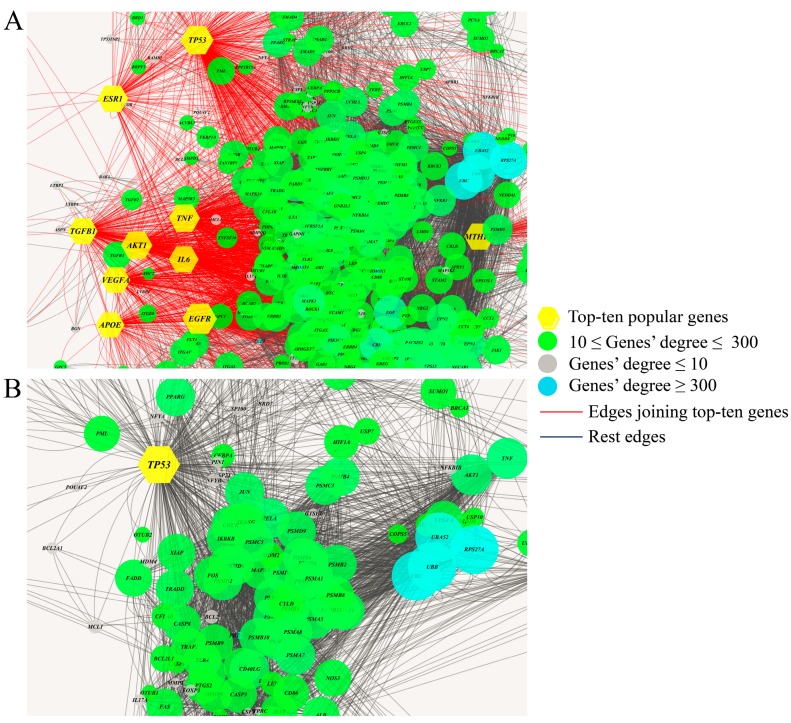
The network of ten popular disease genes and their first-order neighbor genes. (**A**) The top-ten popular genes and their nearest neighbors. (**B**) TP53 and its neighbor genes.

**Figure 5:**
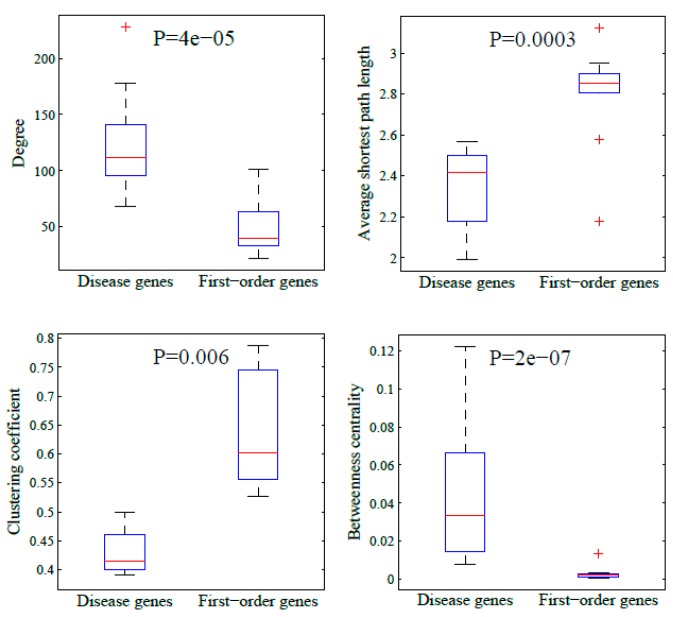
The comparison of the four network parameters in the top-ten popular genes and those in their first-order neighbor genes.

The former parameters indicated the importance of network localization in the ten most popular genes. It is of interest to identify the four parameters of their first-order neighbor genes for their closeness with disease genes. Figure 4 demonstrates the ten genes and their first-order neighbors in the interactome. The boxplots of their topological parameters are shown in Figure 5. For comparison, we also plotted the corresponding parameters of the ten genes. As shown in Figure 5, we found that the network parameters of the first-order genes were distinct with those of the ten disease genes. Compared with Figure 3, we found that the parameter patterns in the first-order neighbor genes were similar to those in the random genes. This proves the disease genes had different topological parameters to their first-order neighbor genes. The result indicates that the disease genes were located in special positions in the interactome and the network localization was a major determinant factor for their malfunctional roles.

### 3.3. The Parameters in the Other Disease Genes

**Figure 6:**
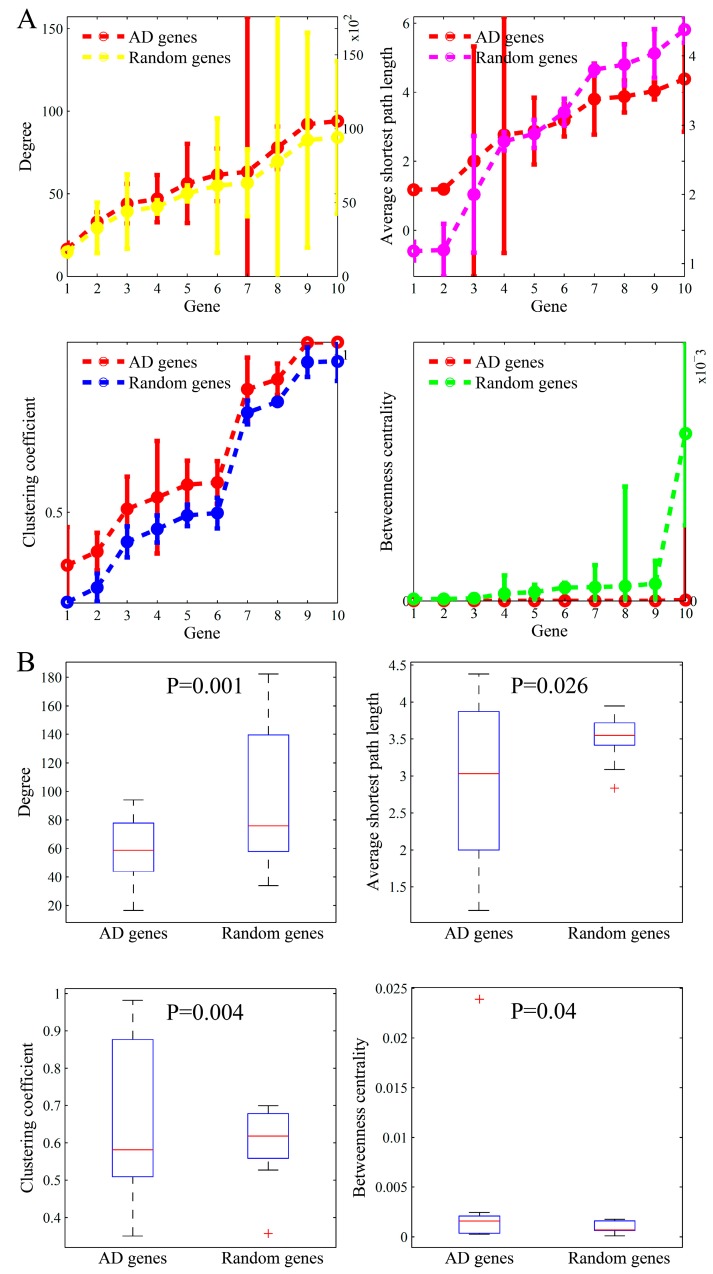
The comparison of topological parameters between AD (Alzheimer’s disease) genes and randomly-selected genes. (**A**) The means (left y-axis) and standard deviations (right y-axis) of the four parameters in the ten AD gene groups and those corresponding in the randomly-selected ten gene groups. (**B**) Boxplots of these parameters.

**Figure 7:**
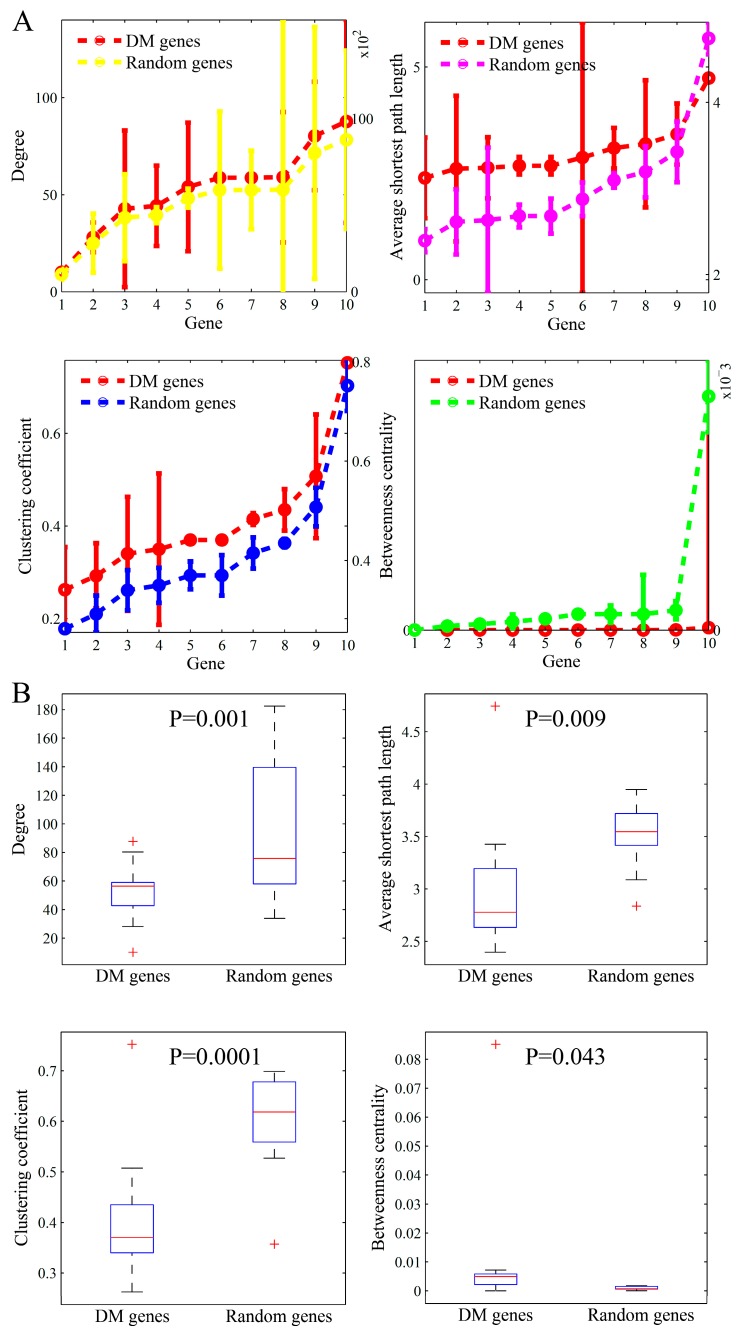
The comparison of topological parameters between DM (diabetes mellitus) genes and randomly-selected genes. (**A**) The means (left y-axis) and standard deviations (right y-axis) of the four parameters in the ten AD (Alzheimer’s disease) gene groups and those corresponding in the randomly-selected ten gene groups. (**B**) Boxplots of these parameters.

**Figure 8:**
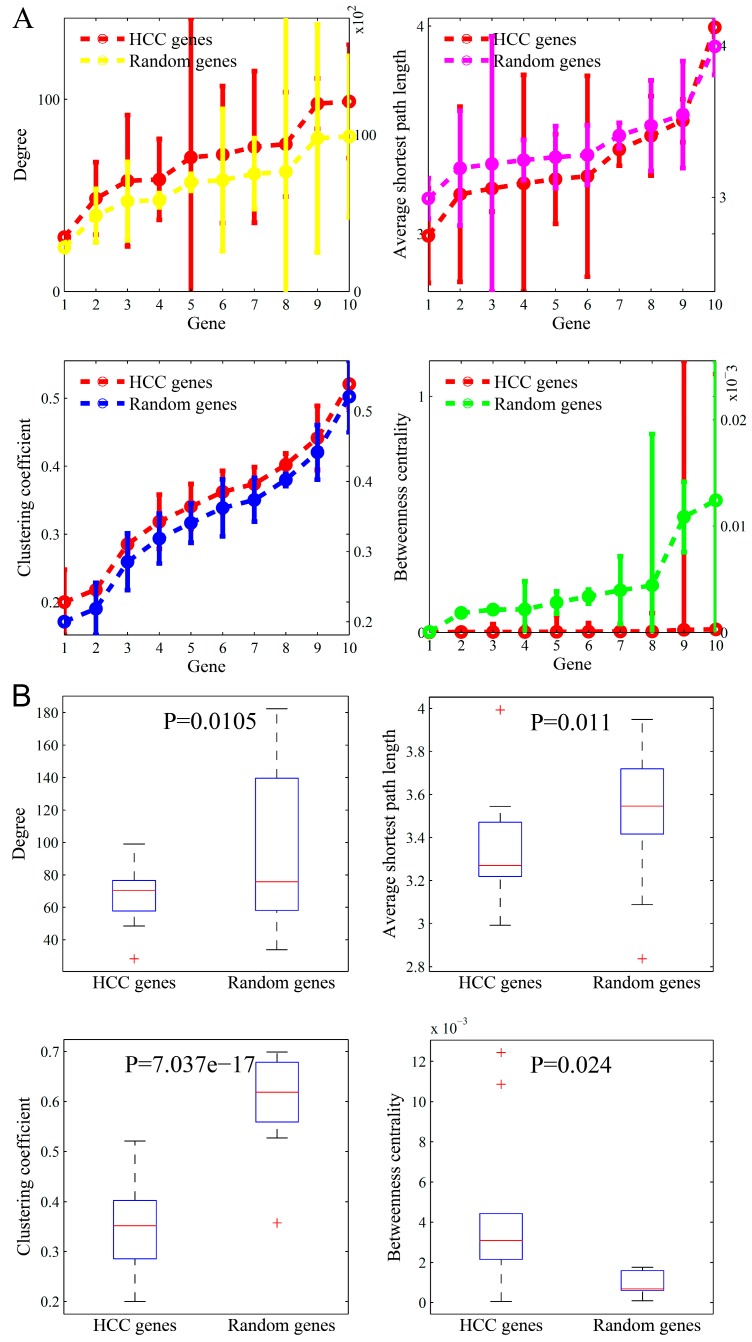
The comparison of topological parameters between HCC (hepatocellular carcinoma) genes and randomly-selected genes. (**A**) The means (left y-axis) and standard deviations (right y-axis) of the four parameters in the ten AD (Alzheimer’s disease) gene groups and those corresponding in the randomly-selected ten gene groups. (**B**) Boxplots of these parameters.

We identified the specific network topological patterns underlying disease genes from the most popular genes. For justifying these findings in particular diseases, we further studied the topological parameters of the other disease-related genes in three major types of complex diseases (i.e., AD, DM, and HCC). For the different numbers of disease genes, we divided these genes into 10 groups with equal sizes in the three diseases. Similar to the ten most-popular genes, we calculated the statistics for the ten-gene groups in the three diseases individually. The results of AD, DM, and HCC are shown in Figure 6, Figure 7, and Figure 8, respectively.

The results in the three figures are presented in parallel manners. As shown in Figure 6, the subfigures plot the node degree, average shortest path length, clustering coefficient, and betweenness centrality of AD genes, respectively. The topological parameters in the same size of randomly selected gene sets are also shown. The results in Figure 7 and Figure 8 are very similarly presented.

From the comparison studies, we found that the results in the three complex diseases were consistent with those identified in the top-ten popular genes, except the parameter pattern about degree. The degree of the top-ten popular genes was significantly higher than that of the random genes, whereas it was marginally higher in the three diseases than in the random genes. Even, the degree median in the three diseases was slightly lower when compared to normal genes. The independent validations from the other diseases mutually justify our findings that the disease genes in the biomolecular network often locate in important positions with special topology parameters. The location in an interactome plays an important role in determining the dysfunction of a gene in complex diseases.

### 3.4. Correlation between Parameters

The four topological parameters are often employed to describe network properties [4]. To investigate their interrelations, it is of interest to check their correlation coefficients. In the same philosophy, we implemented the calculations of parameter correlations for the ten genes and justified the findings in the disease genes of AD, DM, and HCC. The results are shown in the following figures.

**Figure 9:**
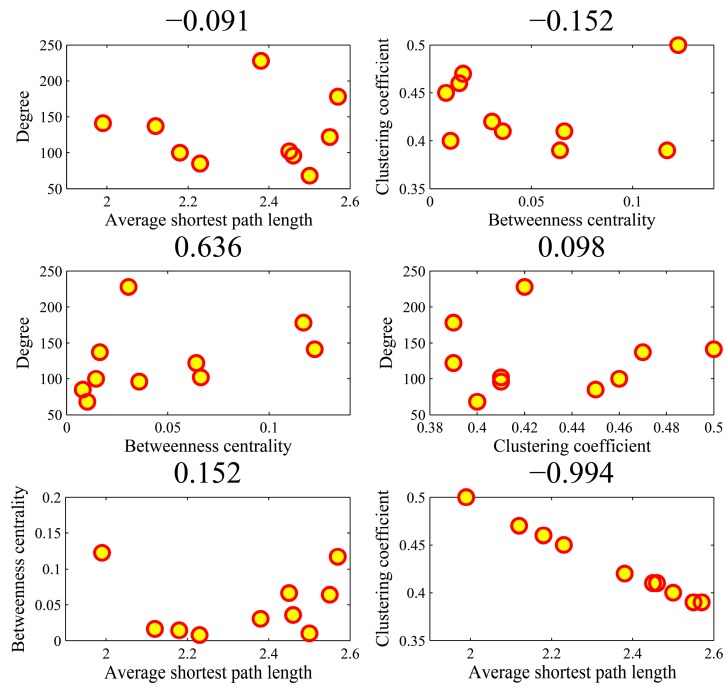
The Spearman rank correlations between the four parameters in the top-ten popular genes.

**Figure 10:**
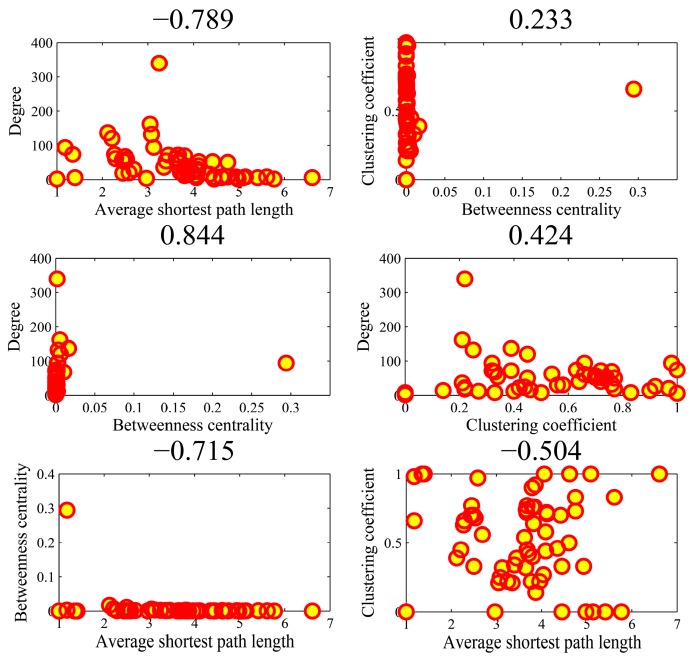
The Spearman rank correlations between the four parameters in the AD (Alzheimer’s disease) genes.

**Figure 11:**
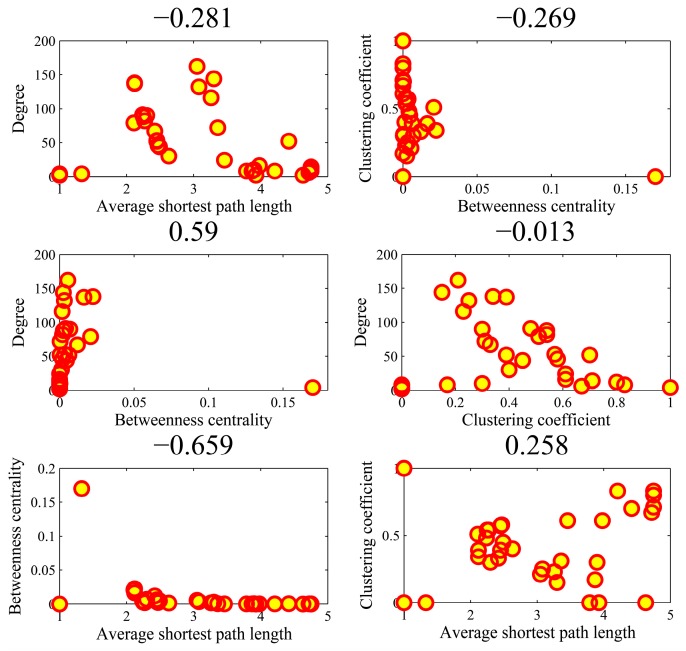
The Spearman rank correlations between the four parameters in the DM (diabetes mellitus) genes.

**Figure 12:**
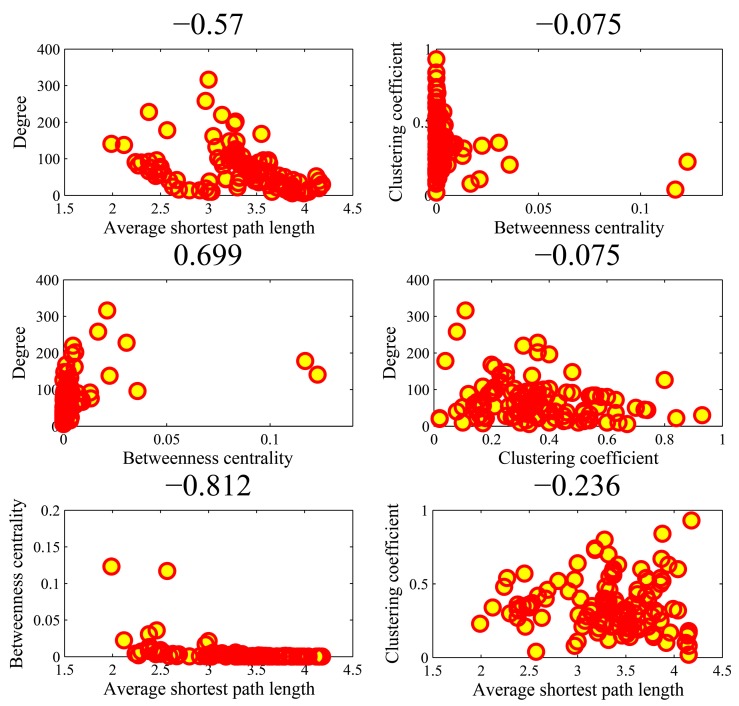
The Spearman rank correlations between the four parameters in the HCC (hepatocellular carcinoma) genes.

Figure 9 illustrates the all-against-all correlations ($C_{4}^{2}=6$) in the four parameters for the top-ten popular genes. We found that the association between average shortest path length and clustering coefficient achieved an outlier coefficient of −0.994. The high correlation value indicated the consistency between the rankings of two topologies in the ten genes. There were no high correlations between the other pairs of four parameters. Figure 10 shows the correlations in the AD genes. The correlation between degree and betweenness centrality was as high as 0.844. The other pairs could not obtain higher correlations. In the cases of high correlation values of 0.844 and −0.715, the betweenness centrality only achieved low values. As shown in Figure 11 for DM genes, the correlation between average shortest path length and betweenness centrality achieved the highest value of −0.660. The relationship between degree and betweenness centrality was rather high at 0.590. In HCC genes shown in Figure 12, the correlation between average shortest path length and betweenness centrality achieved the highest value of 0.812. The outlier points in each subfigure also had no special corresponding rules between any two parameters. The results provide evidence that these parameters were relatively independent, except in a few isolated cases. Thus, they can be employed to represent the location properties in the interactome. They have specific meanings and references in describing network topologies. An interesting research direction is to investigate the differences between these network topologies and check their abilities in representing network localization. We employed all the four parameters to describe the network topology.

## 4. Conclusions

The outcome of complex diseases is contributed to from the dysfunctional interactions of multiple genes, molecules, and the environment. The complex mechanisms of disorder often bring tremendous difficulties in the prevention, diagnosis, and treatment of complex diseases. The network techniques provide powerful tools of organizing the functional relationship and structure via comprehensive interactome. In this philosophy, the network topological parameters are important to describe the network. In this work, we provide a study of investigating the network properties of disease genes in an interactome by integrating functional linkages among genes. The findings highlight the importance of location in the network for complex disease genes.

We firstly investigated the network parameters in the top-ten most popular disease genes. We found that the parameters of them are often different from those of the normal genes. This indicates the location of network is related to the dysfunctions of the complex diseases. We then confirm our findings in the disease gene sets of three major complex diseases (i.e., AD, DM, and HCC). Similar topological patterns in these disease genes were observed with those of the top-ten popular genes. We justified our results of topological importance of the disease genes in the current uncomplete interactome. The parameters were relatively quantified via the same background of interactome. When more gene interactions and context-specific networks become available, the renewed parameters will demonstrate the changing dynamics of network topology.

To further check the importance of network localization for disease genes, we studied the topological features in their nearest neighbors. Their network topological patterns were also different from those of disease genes. In order to further confirm the meaningfulness of network parameters, we identified pairs of correlations between parameters. The analyses demonstrated most of them had no significant correlation to each other. Thus, the characteristics of independent network property can be used to partially describe the network topology.

Our results and findings imply the locations of disease genes in interactome play crucial roles in disease onset and progression. The network topology indicates the pathogenesis of complex diseases. From the network perspective, it is possible to deepen our understanding of the functionality transmission between genes and decipher novel mechanisms of complex diseases. Our analysis reveals the importance of investigating the topological structures of disease genes from the network perspective in systems biomedicine.

## Figures and Tables

**Table 1:** The top-ten most popular genes.

Symbol	Gene ID	Function
*TP53*	7157	Associated with the tumor suppressor protein p53. Mutation causes more than half of human cancers.
*TNF*	7124	An immune molecule associated with tumor necrosis factor, as a major drug target in inflammatory diseases.
*EGFR*	1956	Associated with the epidermal growth factor receptor, a membrane-bound receptor protein. Mutations may lead to drug-resistant cancer.
*VEGFA*	7422	Associated with vascular endothelial growth factor A, helping promote the growth of blood vessels.
*APOE*	348	Associated with cholesterol and lipoprotein metabolism, namely apolipoprotein E.
*IL6*	3569	Associated with an immune molecule namely interleukin 6, helps to stimulate and suppress inflammation.
*TGFB1*	7045	Associated with transforming growth factor β 1, used to regulate cell proliferation and differentiation.
*MTHFR*	4524	Associated with process amino acids, an enzyme named methylene-tetrahydrofolate reductase.
*ESR1*	2099	Associated with a nuclear receptor protein, estrogen receptor 1, which plays an important role in breast, ovarian, and endometrial cancers.
*AKT1*	207	Associated with a signaling protein namely kinase, helping activate other proteins by phosphorylation.

**Table 2:** The values of network topology parameters in the top-ten popular genes.

Gene	Topology Parameter			
	Degree	Average Shortest Path Length	Clustering Coefficient	Betweenness Centrality
*TP53*	178	2.57	0.39	1.17 × 10^−1^
*TNF*	137	2.12	0.47	1.64 × 10^−2^
*EGFR*	228	2.38	0.42	3.06 × 10^−2^
*VEGFA*	85	2.23	0.45	8.05 × 10^−3^
*APOE*	68	2.50	0.40	1.03 × 10^−2^
*IL6*	100	2.18	0.46	1.45 × 10^−2^
*TGFB1*	96	2.46	0.41	3.59 × 10^−2^
*MTHFR*	102	2.45	0.41	6.63 × 10^−2^
*ESR1*	122	2.55	0.39	6.41 × 10^−2^
*AKT1*	141	1.99	0.50	1.23 × 10^−1^

## Supplementary Materials

The following are available online at https://www.mdpi.com/2073-4425/10/2/143/s1, Supplementary material S1: Disease gene lists for Alzheimer’s disease, diabetes mellitus, and hepatocellular carcinoma.
